# Effects of context on the neural correlates of attention in a college classroom

**DOI:** 10.1038/s41539-021-00094-8

**Published:** 2021-07-06

**Authors:** Jennie K. Grammer, Keye Xu, Agatha Lenartowicz

**Affiliations:** 1 School of Education and Information Studies, University of California, Los Angeles, CA USA; 2 Semel Institute for Neuroscience and Behavior, Department of Psychiatry & Biobehavioral Sciences, University of California, Los Angeles, CA USA

**Keywords:** Neuroscience, Education, Human behaviour

## Abstract

Activities that are effective in supporting attention have the potential to increase opportunities for student learning. However, little is known about the impact of instructional contexts on student attention, in part due to limitations in our ability to measure attention in the classroom, typically based on behavioral observation and self-reports. To address this issue, we used portable electroencephalography (EEG) measurements of neural oscillations to evaluate the effects of learning context on student attention. The results suggest that attention, as indexed by lower alpha power as well as higher beta and gamma power, is stronger during student-initiated activities than teacher-initiated activities. EEG data revealed different patterns in student attention as compared to standardized coding of attentional behaviors. We conclude that EEG signals offer a powerful tool for understanding differences in student cognitive states as a function of classroom instruction that are unobservable from behavior alone.

## Introduction

Online measures of student attention have long been of interest to educators seeking to promote student learning^1,2^. Understanding individual differences in student attention and the classroom activities that can support engagement has important implications for academic success^3–5^. However, field studies of attention in the real-world setting of the classroom are limited. This is in large part because measuring attention in real-world settings is challenging. Methods commonly employed to assess attention during learning— including self-report^6–8^, behavioral observation^1,9^, and assessment of learning-related activities and outcomes^10^—are each indirect and cannot pick up the dynamic changes in student engagement. As a result, these tools are limited in the extent to which they capture individual differences and fluctuations in attention during instruction^11^. There exists a need, therefore, for new methodology for objective real-time assessment of attention in the classroom setting.

Advances in portable EEG technology have made it possible to collect high-quality data in real-world settings. These methods have recently been used to explore student’s cognitive processes in the classroom^12,13^. In the current study, we leverage the high signal-to-noise ratio, strong mechanistic bases, and clear functional association of “alpha” range (8–12 Hz) neural oscillations with attention system functionality^14–16^, to quantify attentional engagement of undergraduate students. Decreases in alpha power (i.e., squared magnitude of the oscillations) have been extensively associated with engagement of attentional circuitry^17^, and increased perception, visual attention and encoding^18^. Here, we measure alpha power in EEG data collected from multiple participants concurrently engaged in classroom instruction during a college lecture across four types of instructional context typical of college classrooms (instructor initiated: lecture, video watching; student initiated: group work, and independent work) and compare these data with video-based observer ratings of student attentiveness. We hypothesized that passive activities (lecture and video watching) would be less engaging, and thus should show greater indices of inattention, than interactive activities (group and independent work). In doing so, we demonstrate the benefits of EEG methods for assessing attention in real-world settings, which provide access to understanding cognitive processes that are difficult for teachers to observe directly.

## Results

### EEG as a function of instructional context

**Fig. 1:**
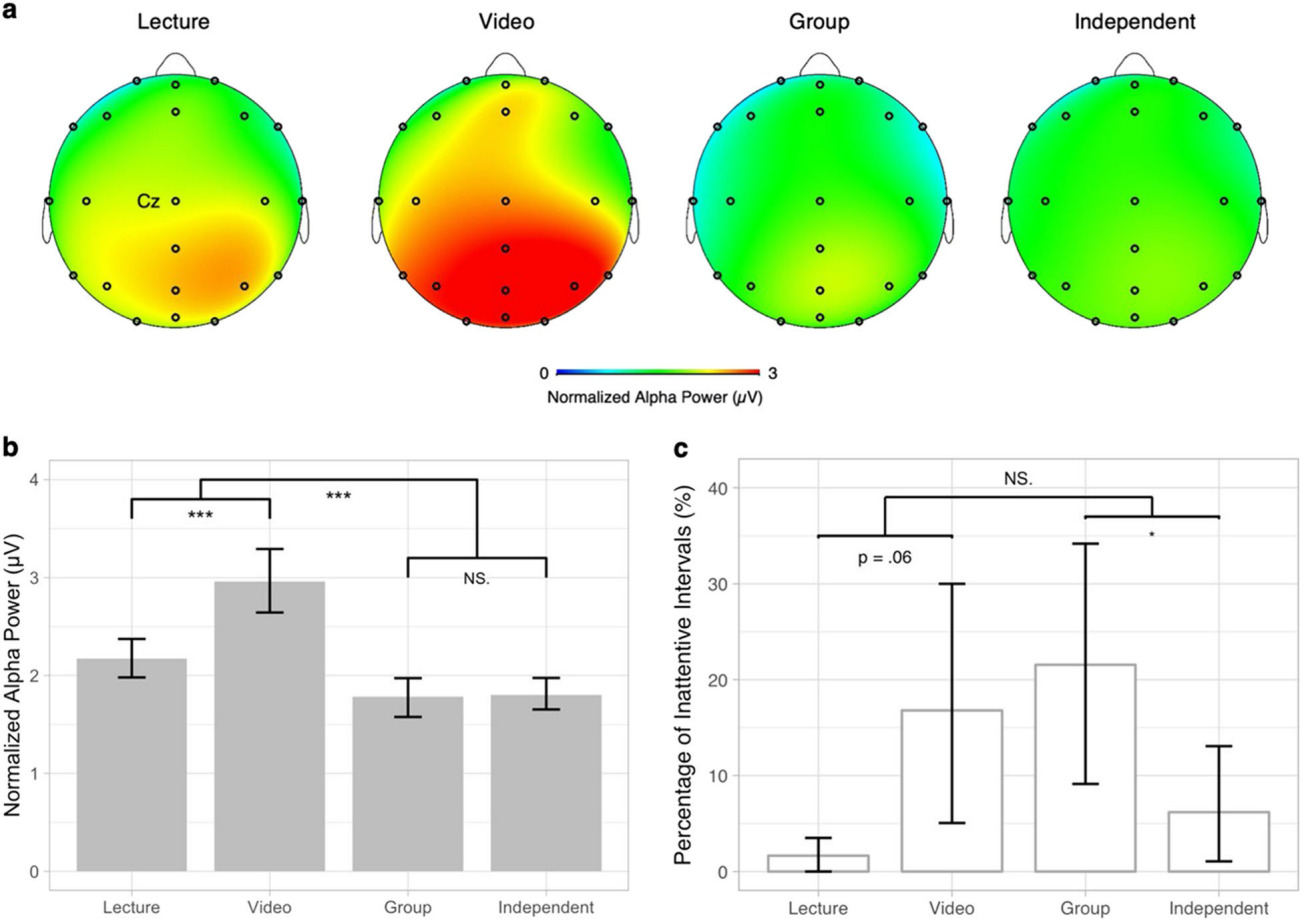
Effects of instructional activities on student attention as indexed by normalized alpha power and observer rating. **a** Topographic maps for alpha power revealed that alpha power was higher during lecture and video watching and had a posterior scalp distribution. **b** Student attention was measured as mean normalized alpha power for each instructional activity, with error bars representing 95% confident interval (calculated by bootstrapped standard error). **c** Student attention was rated by trained observers as the proportion of inattentive intervals over all codable intervals in each activity, with error bars representing 95% confident interval (calculated by bootstrapped standard error). Same planned contrasts were performed for measures of alpha power and observer rating portrayed in **b** and **c**, *p < 0.05, **p < 0.01, ***p < 0.001.

Student attention during lectures, as indexed by normalized alpha power, varied significantly as a function of instructional activity type (*χ*^2^(3) = 41.39, *p* < 0.001). Alpha power over the occipital cortex (see Fig. 1a) was greatest, supporting the inference that students were least attentive, during video watching (see Fig. 1b). Planned contrasts revealed that alpha power was significantly higher, consistent with lower student attention, in teacher-initiated activities (lecture and video watching) than in student-initiated activities (group work and independent work), *b* = −0.43, *t* (55) = −6.32, *p* < 0.001, *r* = 0.65. Students were also found to be significantly more disengaged while watching the video (*M*_Video_ = 2.96) than when listening to the lecture (*M*_Lecture_ = 2.17, *b* = −0.41, *t*(55) = −4.47, *p* < 0.001, *r* = 0.52), whereas no differences were observed between student-driven group work (*M*_Group_ = 1.78) and independent work (*M*_Independent_ = 1.80, *b* = 0.01, *t*(55) = 0.14, *p* = 0.89, *r* = 0.02).

Widely cited anecdotal reports indicate that student attention is limited to 10–15 min, changing dynamically and increasing during activity transitions^7,11^. We examined this claim by comparing alpha power across activities for the 2 min at the start versus the end of each 10 min-activity. Alpha power was significantly lower during the starting period (*M*_Start_ = 2.08) of the activity then the ending period of each activity (*M*_end_ = 2.20), *χ*^2^(1) = 7.81, *p* = 0.005, and showed a significant interaction effect with activity type, *χ*^2^(3) = 14.93, *p* = 0.002. Further comparisons indicated that alpha was significantly lower in the starting 2 min than the ending 2 min during the independent work only, *b* = −0.43, *t* = −3.72, *p* < 0.001, *r* = 0.21, suggesting that time-related differences in student attention only occurred when students finished work independently.

### Student behavior

We also compared student attention quantified by observer ratings, the dominant metric used to examine attention in real-world settings. As seen in EEG data, observational data revealed a significant effect of activity type on students’ inattentiveness, *χ*^2^(3) = 9.30, *p* < 0.05. Consistent with neural measures, students were rated as showing marginally higher level of inattentiveness in video watching (*M*_Video_ = 17%) compared to lecture (*M*_Lecture_ = 2%, *b* = −0.07, *t*(41) = −1.96, *p* = 0.06, *r* = 0.29). In contrast to neural measures, there were no observable differences in teacher-initiated relative to student-initiated activities (*b* = −0.02, *t*(41) = −0.71, *p* = 0.48, *r* = 0.11), and attention was rated as lower during the group (*M*_Group_ = 22%) relative to independent work (*M*_Independent_ = 6%, *b* = 0.08, *t*(41) = 2.28, *p* = 0.03, *r* = 0.34).

## Discussion

In the current study, we directly linked classroom experience to brain function to examine the ways in which classroom experience impacts student attention. Neural measures of attention differed significantly across instructional activities, revealing that attention was highest during student-initiated activities, followed by lecture. Despite student’s preference for videos incorporated in classroom lecture, our data also suggest that this type of classroom activity provides opportunities for disengagement that could have implications for learning.

Oscillatory EEG signals recorded over visual cortex are regarded as one of the most stable of human oscillatory patterns and have been associated with attentional processes^18^. Extensive evidence indicates that increases in alpha power are associated with disengagement of attention^14,18^ and weakened alpha modulation has been linked to ADHD^16,19^. The use of alpha signal as an index of attention has been further validated in concurrent EEG-fMRI^16,20^ studies, that have linked this signal to occipital, thalamic and fronto-parietal activation, all substrates supporting visual attention. It is notable, that alpha power also increases any time that visual sensory input decreases, such as when the eyes are closed or when auditory inputs dominate^14,18^. This alternate explanation is unlikely in the current study, however, because light levels were comparable, and eyes were open across all conditions. Arguably visual stimulation was elevated for video presentation, which showed the highest rather than the lowest alpha power. Furthermore, supplemental analyses (c.f., SI) additionally reveal that teacher-initiated activities also increased theta-band (4–7 Hz) power and decreased both beta (13–20 Hz) and gamma (30–50 Hz) power, consistent with a neural shift from higher to lower oscillations, typical of transitions to lower wakefulness^21,22^. This offers convergent evidence for the interpretation of the results as consistent with a shift in visual attention. However, we note that the current approach would require further study in learning context dominated by auditory content, as alpha modulation in auditory attention has been less consistently assessed^18^.

Broadly, the goal of this work is to help teachers better understand the behavioral manifestations of attention in the classroom, and this investigation represents a first step in our efforts to examine the feasibility of using EEG to examine unobservable cognitive states in naturalistic classroom settings. Notably, neural data revealed a different, more nuanced story than was provided by behavioral coding alone. This direct contrast was particularly apparent in divergent conclusions drawn from behavioral ratings versus EEG data during lecture versus group work. Students were more frequently observed looking away during group work than during lecture, contributing to behavioral ratings identifying the former as more inattentive. However, EEG data suggested the opposite conclusion, which may indicate that these instances during group work might be periods of attentional engagement instead of distraction.

Few studies have examined EEG in real-world classroom settings and, of those, fewer still have linked aspects of instruction to student’s cognitive states^12,23^ and student learning^23,24^. It is important to note that some results we report here—namely that alpha power was greatest during video viewing—differ from those found in two similar investigations^13,25^. Although the reasons for this divergent pattern of findings are unclear, it is likely that differences in EEG methodology and in the nature of experimentally manipulated instruction were contributing factors. In terms of methods, in contrast to other recent studies involving classroom-based EEG^13,25^, our EEG measures do not include inter-brain synchrony. Issues of data loss and data quality have emerged as a key limitation and concern in real-world neuroscientific research^26^. In contrast to previous studies, in this investigation we used research-grade EEG equipment resulting in limited loss across conditions, which could have impacted our results. In addition, the instructional activities were standardized and materials—including the video and lesson content—were drawn directly from existing undergraduate lectures. We suspect that differences in instructional materials, content, and duration of lessons play an important role in student engagement and see this as a rich area for future research. More research is also needed to understand how neural measures of attention relate to student outcomes. Similarly, convergent measures of student attention—including self-report—would further clarify the ways in which EEG measures relate to behavior.

Although we were unable to counterbalance activity type and observed students in only one session in this investigation, notably, activities where neural measures of attention were highest occurred during the last half of the lessons, suggesting that students growing bored or fatigued across activities did not account for the patterns observed. These results also highlight that interpretation of behavioral cues (e.g., looking away) may be more nuanced than previously reported^1^. Furthermore, behavioral observations were more subjective and error prone than the EEG metrics, suggesting that observations yield less reliable measurement. This provides evidence that the behavioral cues relied on to evaluate student attention—which are limited to gaze and overt motor movements—may be less sensitive to covert processes associated with attention. Through years of experience in school, students are socialized in behaviors that reflect positive approaches to learning, such as making eye contact, potentially resulting in inaccurate perceptions of student engagement on the part of the instructor. Given the challenges associated with using behavioral and report measures of attention^2^, these results are particularly promising for understanding cognitive processes that are difficult to observe directly in the real-world.

In conclusion, leveraging portable EEG neural measures to examine attention in naturalistic settings, we have not only demonstrated differences in attention as a function of classroom activity, but we have also observed individual differences in attention across students concurrently experiencing the same lessons. This work contributes to a growing body of research demonstrating the feasibility of using EEG to examine the covert processes of attention in the classroom setting using naturalistic and task-based^12^ paradigms. Further research on the types of activities that best support attention for individual learners will allow us to make evidence-based suggestions to teachers about how to structure classroom instruction.

## Methods

### Sample

A diverse group of 23 healthy college students (*N*_men_ = 5, ages 18 to 23 years old) from a large public university participated (35% White, 17% Latinx, 39% Asian, and 8% other). Six participants reported speaking another language than English growing up. Written informed consent was obtained from each participant in advance of the study in accordance with the University of California, Los Angeles Office of the Human Research Protection Program (OHRPP) approval.

### Procedure

Data were collected in a college classroom where students participated in a lesson on educational neuroscience taught by a graduate teaching assistant. Students participated in groups of 4–9. In total eight groups of students participated. From each group, two to three students were randomly selected to be assessed with portable EEG. Lessons consisted of four 10–15-min instructional activities reflective of those common in a college course, taught in the following order: (1) whole-group lecture, (2) video watching, (3) group discussion, and (4) independent work. Sociodemographic data were collected for all of the participants and lessons were video-recorded.

### EEG data recording, reduction, and analysis

EEG was recorded using the SMARTING mobile EEG amplifiers (mBrainTrain, Belgrade, Serbia) with 24 Ag/AgCl active scalp electrodes. Data were digitized at 250 Hz, with AFz as the ground and FCz as reference during recording, and re-referenced offline to the average of all the scalp electrodes. Electrode impedances were <50 kΩ. Data were examined and analyzed using BrainVision Analyzer 2 (Brain Products, Germany). Data were first screened by visual inspection to remove extreme artifacts. A band-pass filter of 1–50 Hz using 0-phase shift was then applied, followed by screening using automated algorithms that marked epochs in which (a) voltage step changes exceed 50 mV/ms or (b) absolute voltage exceeded 300 mV or (c) peak-to-peak activity was greater than 500 mV within 200 ms or (d) maximum voltage difference less than 0.5 mV within a trial. Ocular movement artifact correction was conducted using a regression-based algorithm in BVA and FP1/FP2 were used as eye-movement indicators.

EEG data were then segmented based into four types of instructional activity and within each segment, continuous EEG signal were further segmented into 20 s-length epochs with 50% overlapping between two consecutive epochs. Epochs that were marked as bad were deleted during this step and participants with >40% EEG data eliminated due to artifacts were not included in the EEG analysis (*N* = 2). For detailed information regarding EEG data loss in each instructional activity, see Supplementary Table 2.

A band of 7.5–12.5 Hz was determined as the alpha frequency band for the study based on previous literature. Alpha power value was calculated using the mean power value for each spectrum range, normalized by the global power (1–50 Hz range) per channel. For additional information regarding power at beta, gamma, and theta as well as alternative processing procedures, please refer to Supplementary Results Figs. 1–10.

### Behavioral coding for lesson videos

Behavioral indicators of attention were coded for those participants in EEG assessments. Video tapes of each instructional session were dual coded by trained and reliable coders (*N*_coders_ = 5), with each participant being the focus of an individual coding pass. Inter-rater reliability was calculated using an agreement, average-measure intra-class correlation (ICC) and the resulting ICC was in the excellent range (ICC = 0.97). Although rare, in instances where coders disagreed, decisions were made by consensus. Reliability was obtained coding to master videos. The coding scheme was modified from that which was developed by Rapport and colleagues^4^. Attention was coded into three categories: Attentive, Inattentive, or Missing, reflecting the primary state of participants based on behavioral cues, including body positioning, eye gaze, and activity engagement in 1-min intervals (see Supplementary Table 1). Percentage of Inattentive intervals was calculated as the behavioral indicator of attention for each participant in each activity.

### Statistical analyses

Multilevel linear regressions were used to explore effects of activity on alpha power and behavioral rating of inattentiveness, with the benefit of overpassing sphericity check in conventional repeated-measures analysis of variance. Planned contrasts were subsequently conducted to explore differences as a function of individual activity.

### Reporting summary

Further information on research design is available in the Nature Research Reporting Summary linked to this article.

## Supplementary information

Supplementary Information

Reporting Summary

## Data Availability

The data that support the findings of this study are available from the corresponding author upon request.
