# Supplementary material for: Effects of context on the neural correlates of attention in a college classroom
Source: NPJ Sci Learn. 2021 Jul 6;6:15. doi: 10.1038/s41539-021-00094-8 (PMC8260629; doi:10.1038/s41539-021-00094-8)

Supplementary Information

Behavioral Coding

Supplementary Table 1. Attention codes used in behavioral coding

	Attentive	*Inattentive*
**Lecture**	1. Facing the lecturer<br>2. Nodding<br>3. Participating (asking questions or making comments)	Looking away for 10 continuous seconds or longer
**Video Watching**	1. Body is facing towards video<br>2. Eyes are directed towards video	Looking away for 10 continuous seconds or longer
**Group Work**	1. Engaging with partner(s)<br>2. Working towards completing task	1. Passive; idly sitting<br>2. Looking around the room for 10 continuous seconds or longer
**Independent Work**	1. Not talking<br>2. Looking at work	1. Gazing away from task for long periods of time<br>2. Not working on task
**Missing**	Missing was coded when the particpant was not visable to the coder, which was an infrequent occurance.	

EEG Data Summary
Raw EEG data was first visually inspected to remove extreme artifacts and bad electrodes. The average number of deleted electrodes was 1.50 (SD = 1.63, range 0-6) across participants. For, each continuous EEG recording, an average of 0.69% (SD = 1.77%) of data was marked as artifacts by the automated algorithm in BVA. Any epochs containing artifact were removed from statistical analysis, and the mean percentage of removed epochs was 8.95% (SD = 8.97%, range 1.06 - 34.73%). The number of 20s-length epochs used for analysis per condition was presented in Supplementary Table 2. Results from linear models indicated that percent of removed epochs varied significantly as a function of activity type, $\chi^2(3) = 16.54$, $p < .001$. Tukey multiple comparisons were conducted to compare percent of removed epochs in every pair of conditions and showed that significantly more epochs were removed from Group work than other three conditions, *p*s < .05.

Supplementary Table 2. Summary of EEG data in analysis, with standard deviations.

	Lecture	*Video*	*Group*	*Independent*
No. of Epochs Included	92.43 (6.83)	61.71 (8.33)	44.76 (20.93)	56.28 (7.45)
No. of Epoch Removed	4.48(4.02)	5.05 (8.35)	10.71 (13.61)	4.10 (7.66)
% of Epoch Removed	4.56 (3.89)	7.30 (11.38)	24.00 (33.07)	7.64 (12.73)

Supplementary Results
In these analyses we used multilevel linear models to examine both alpha power and behaviorally coded student attention. This approach provided us with a straight-forward and parsimonious way of assessing our focal questions. Given that our design included repeated measurements from the same group of students, it was necessary to account for this in our handling of the data. This approach was chosen because, as compared to regular linear regression models, multilevel linear models have the advantage of handling the problem of residual dependency in the repeat-measured data. Moreover, when compared to the conventional repeated-measures ANOVA approach, the multilevel approach sets loose the assumption of sphericity in the data and no more corrections (e.g., Greenhouse-Geisser) for *p*-value are required when sphericity is violated. Instead of F-statistics, likelihood ratio ($\chi 2$) was used in this multilevel approach to indicate whether instructional activity significantly predicted EEG power or behavioral measures of attention during the lesson.

In addition to the analyses presented in the manuscript, we also compared the effect of activity types on other EEG oscillation bands, including theta (4-7hz), beta (13-30hz), and gamma (30.5 - 50hz). Additionally, to rule out the possibility that our alpha results were sensitive to the normalization method used in preparing the data, we used two different normalization methods, global normalization (1-50hz) and gamma normalization (30-50hz), which are reported here for each oscillation band (with the exception of the result for globally normalized alpha power, which can be found in the main article). These results, including topographic maps, spectrum plots, and related statistical analyses are reported in Supplementary Figures 1-10 below. All comparative analyses were conducted using the same planned contrasts and methods described within the article, and analyses were done on four electrode sites as reported in the main article, Pz, POz, O1 and O2.

Results using global normalization (1-50hz)

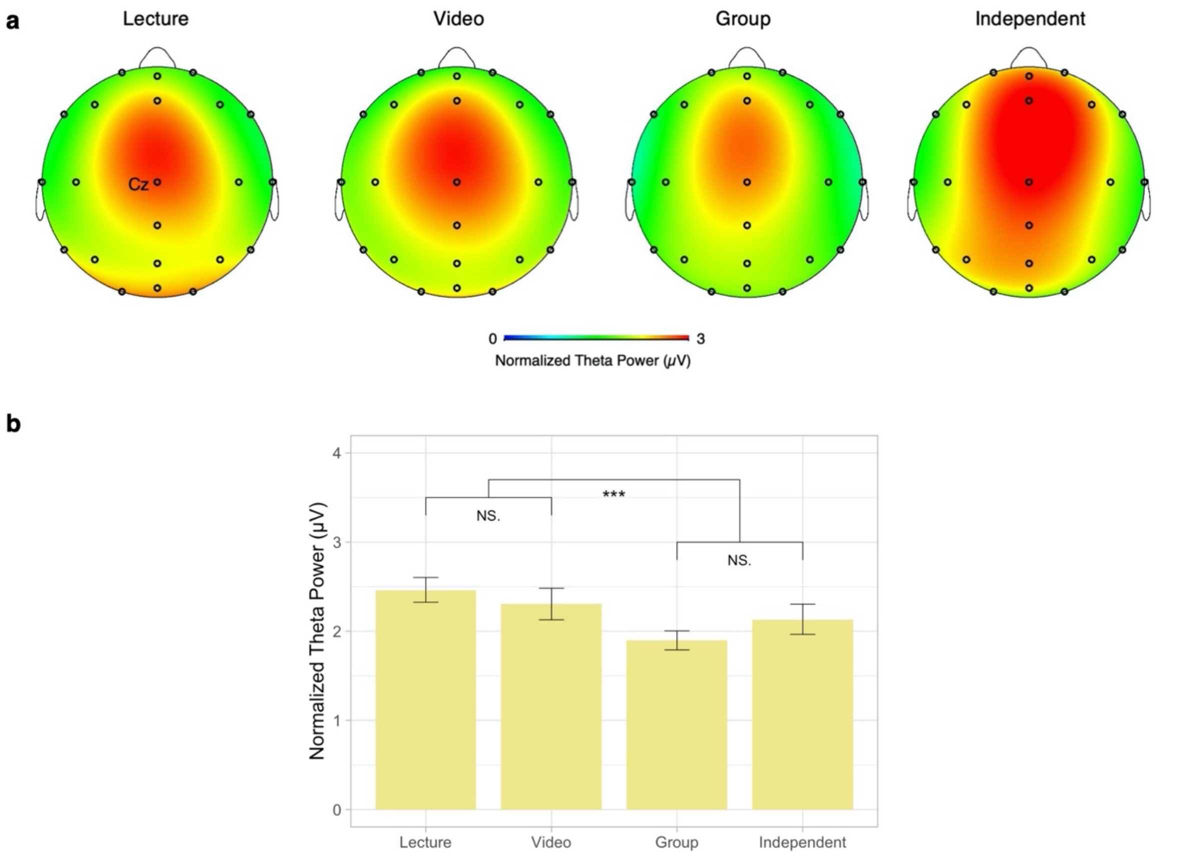

Supplementary Fig. 1: Effects of class activities on theta power (4-7Hz), normalized by global power (1-50hz). **a** Topographic maps of theta band. **b** Histogram of mean theta power in four activities, with error bars representing 95% confident interval (calculated by bootstrapping).

Results showed that instructional activity had a significant effect on theta power, $\chi^2(3) = 14.27$, $p = .003$. Planned contrasts revealed that theta power was significantly lower in student-initiated activities (group work and independent work) than in teacher-initiated activities (lecture and video watching), b = - .17, t (55) = -3.38, $p = .001$. However, no significant difference was found in theta power between lecture and video, nor between group work and independent work (*p*s > .1).

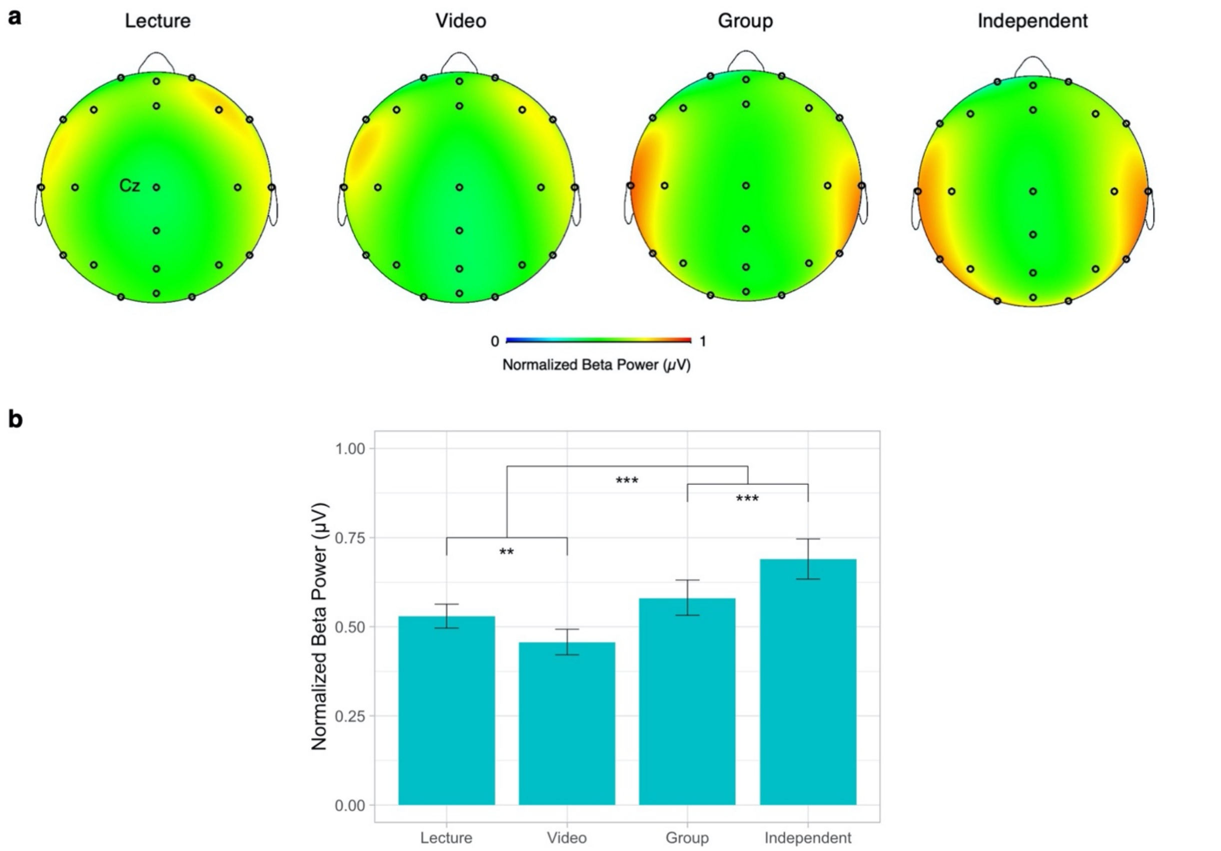

Supplementary Fig. 2: Effects of class activities on beta power (13-30Hz), normalized by global power (1-50hz). **a** Topographic maps of beta band. **b** Histogram of mean beta power in four activities, with error bars representing 95% confident interval (calculated by bootstrapping).

Results showed that instructional activity had a significant effect on beta power, $\chi^2(3) = 60.00$, $p < .001$. Planned contrasts revealed that beta power was significantly higher in student-initiated activities (group work and independent work) than in teacher-initiated activities (lecture and video watching), b = 0.08, t (55) = 8.74, $p < .001$. In addition, beta power was significantly higher during lecture than in video watching, b = .04, t (55) = 3.17, $p = .003$, and lower in group work than in independent work, b = -.06, t (55) = -4.48, $p < .001$.

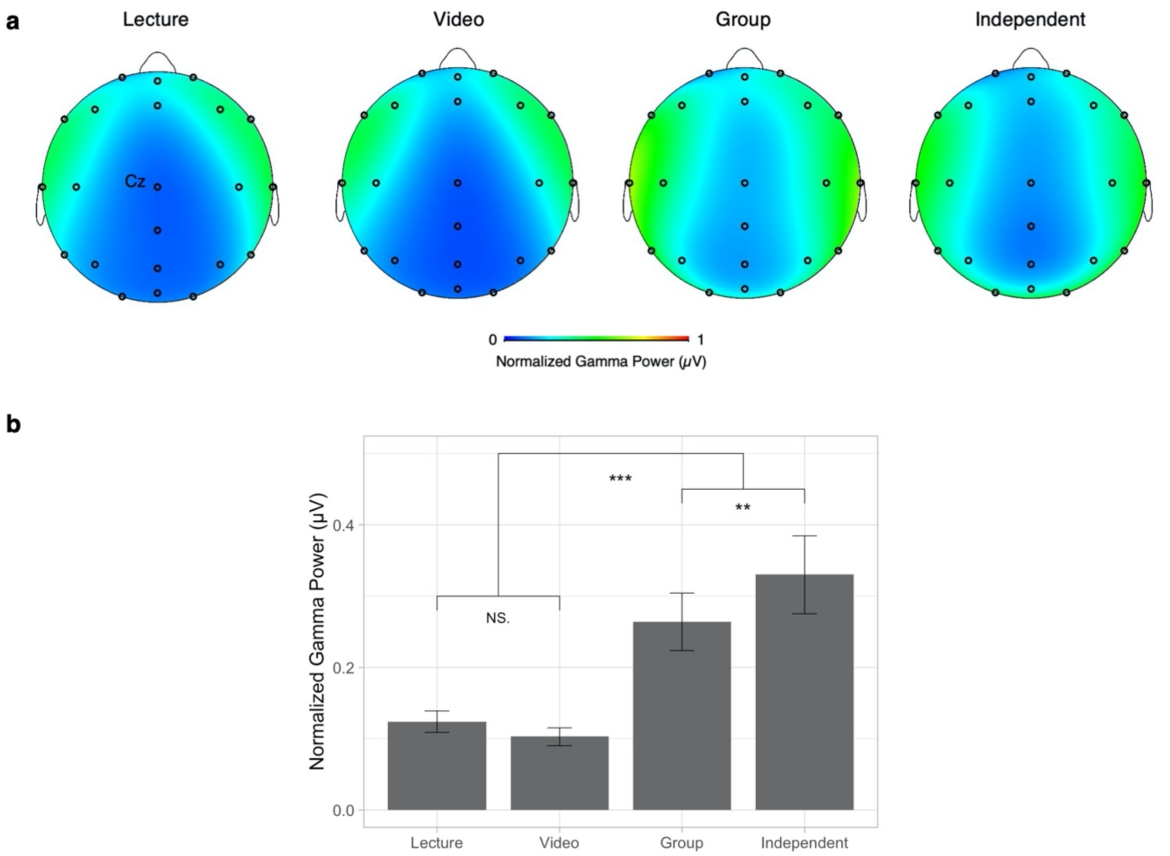

Supplementary Fig. 3: Effects of class activities on gamma power (30.5-50hz), normalized by global power (1-50hz). **a** Topographic maps of gamma band. **b** Histogram of mean gamma power in four activities, with error bars representing 95% confident interval (calculated by bootstrapping).

Results showed that instructional activity had a significant effect on gamma power, $\chi^2(3) = 71.29$, $p < .001$. Planned contrasts revealed that gamma power was significantly higher in student-initiated activities (group work and independent work) than in teacher-initiated activities (lecture and video watching), b = .09, t (55) = 11.51, $p < .001$. In addition, gamma power was higher during independent work than in group discussions, b = .03, t (55) = 2.98, $p = .004$, but no difference was found in gamma power between lecture and video, b = .01, t (55) = .97, $p = .33$.

Supplementary Figure 4a: Spectrum plot of 1hz to 30hz after global normalization.

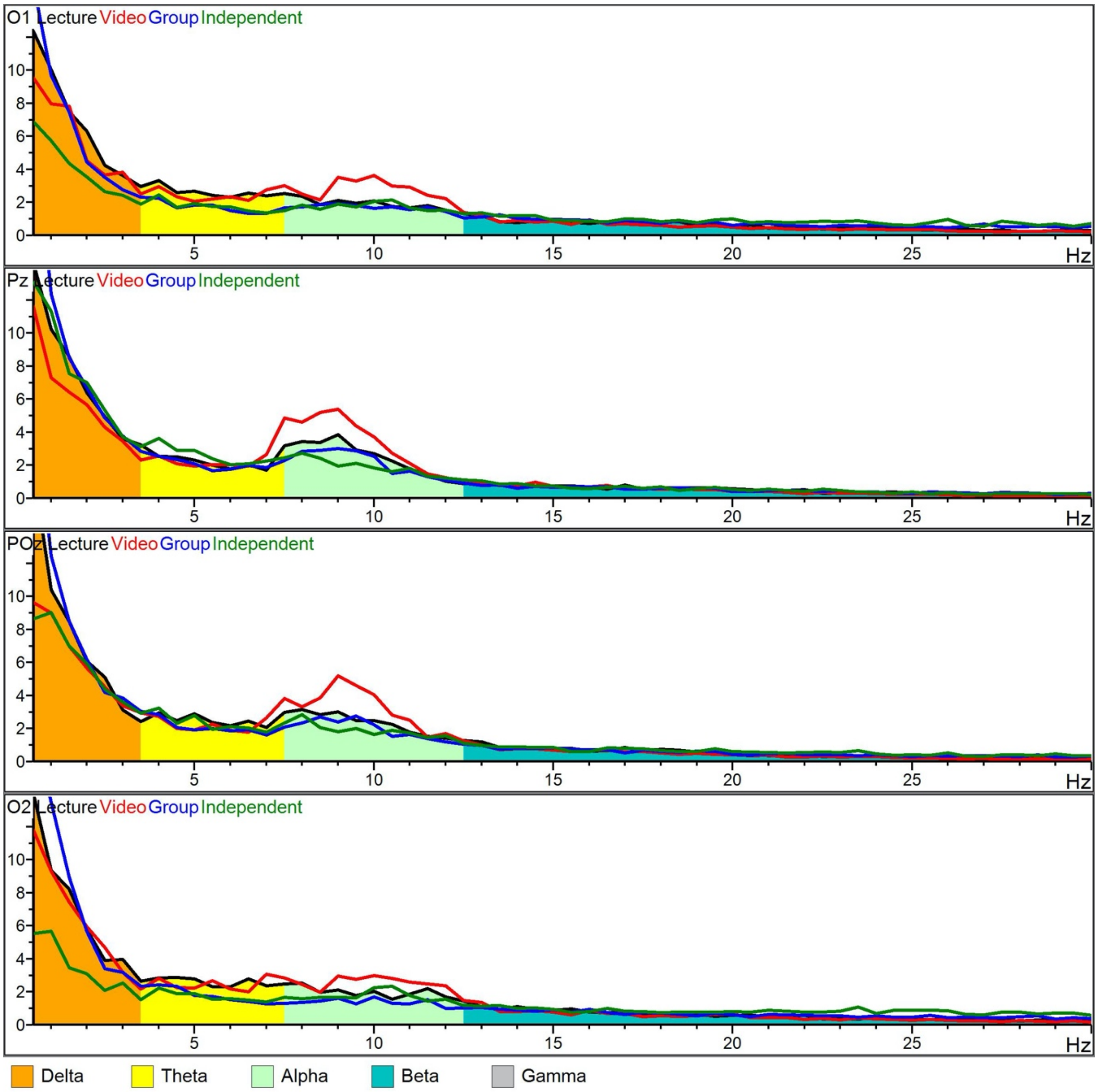

Supplementary Figure 4b: Spectrum plot of gamma band (30.5-50hz) after global normalization.

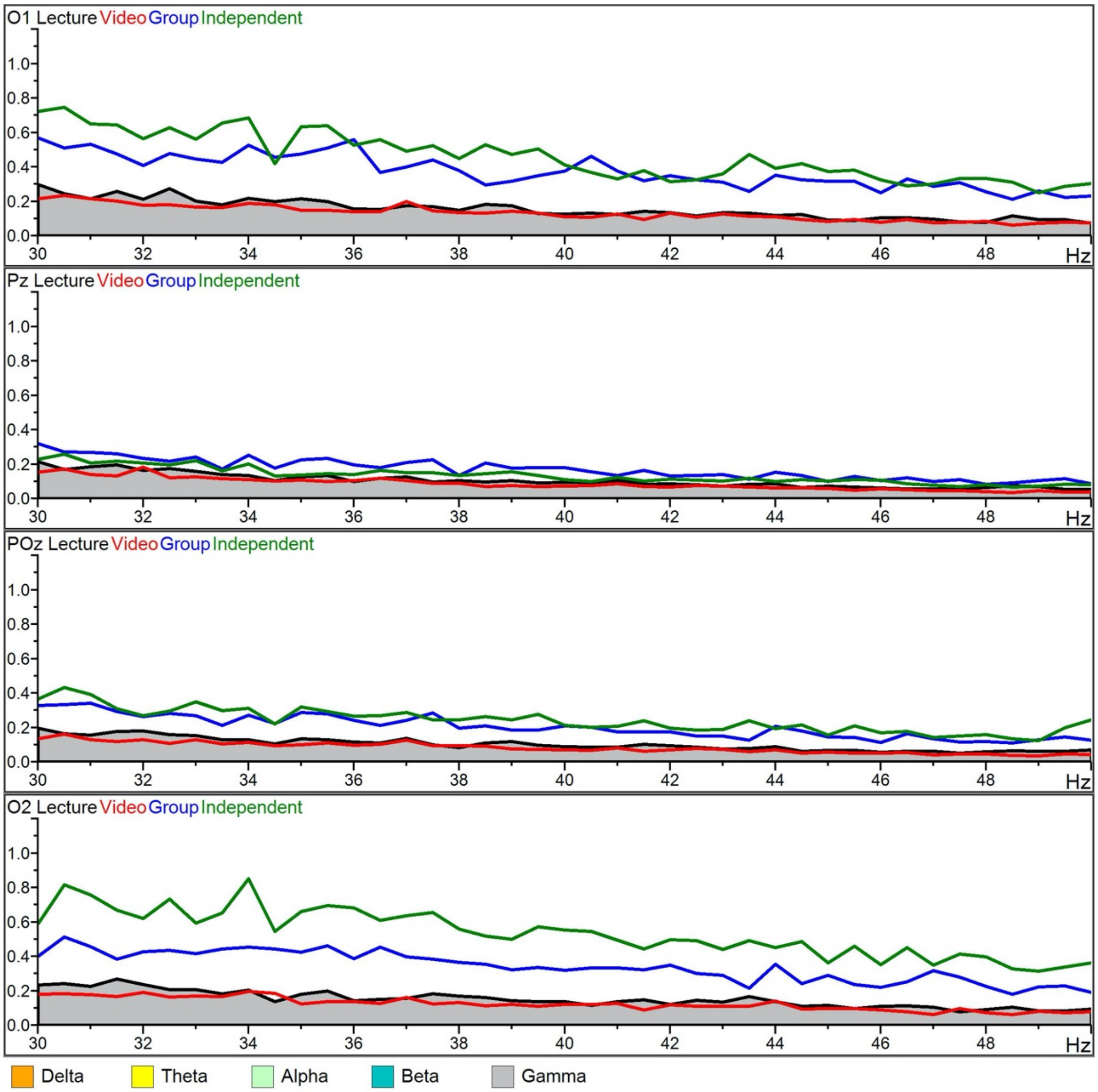

Results using gamma normalization (30-50hz)

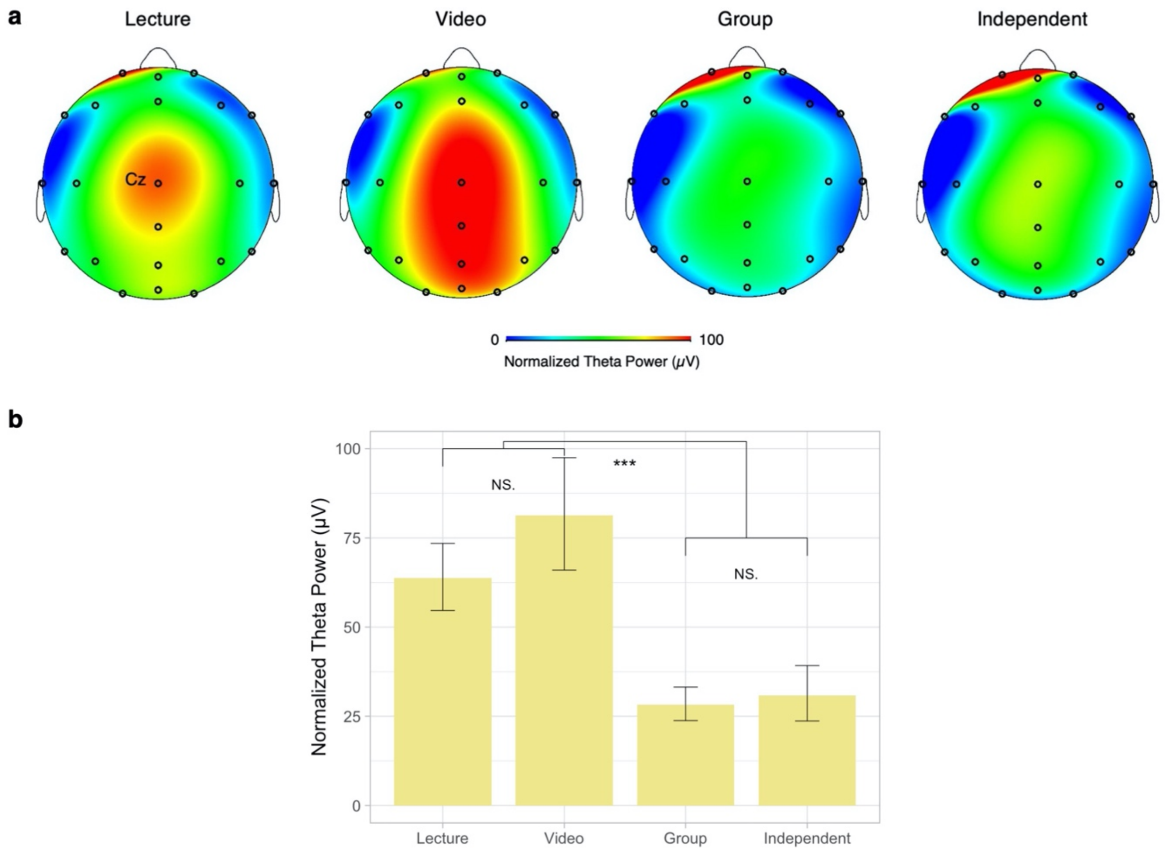

Supplementary Fig. 5: Effects of class activities on theta power (3-7hz), normalized by gamma power (30-50hz). **a** Topographic maps of theta band using gamma normalization. **b** Histogram of mean theta power in four activities, with error bars representing 95% confident interval (calculated by bootstrapping).

Results showed that instructional activity had a significant effect on theta power, $\chi^2(3) = 32.75$, $p < .001$. Planned contrasts revealed that theta power was significantly higher in teacher-initiated activities (lecture and video watching) than in student-initiated activities (group work and independent work), b = 22.56, t (55) = 6.29, $p < .001$. No difference was found in theta power between lecture and video, or between discussion and independent work (*p*s > .06).

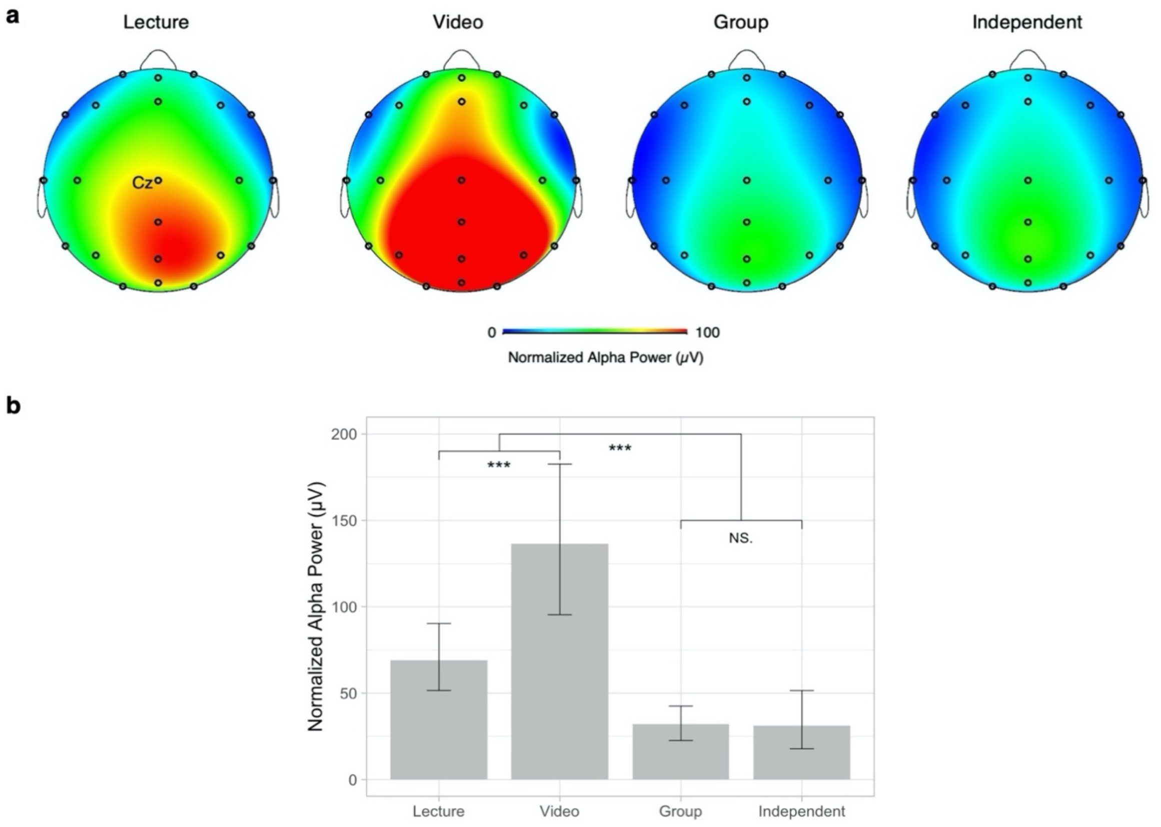

Supplementary Fig. 6: Effects of class activities on alpha power (7.5-12.5hz), normalized by gamma power (30-50hz). **a** Topographic maps of alpha band using gamma normalization. **b** Histogram of mean alpha power in four activities, with error bars representing 95% confident interval (calculated by bootstrapping).

Student attention during lectures, as indexed by alpha power, varied significantly as a function of activity ($\chi^2(3) = 32.04$, $p < .001$). Planned contrasts revealed that alpha power was significantly higher in teacher-initiated activities (lecture and video watching) than in student-initiated activities (group work and independent work), b = -39.33, t (55) = -5.44, $p < .001$. Students were also found to show significantly higher alpha power while watching the video than when listening to the lecture (b = -34.75, t(55) = -3.59, $p < .001$), whereas no differences in alpha power were observed between student-driven group discussion and independent work (b = 1.77, t(55) = .17, $p = .87$)

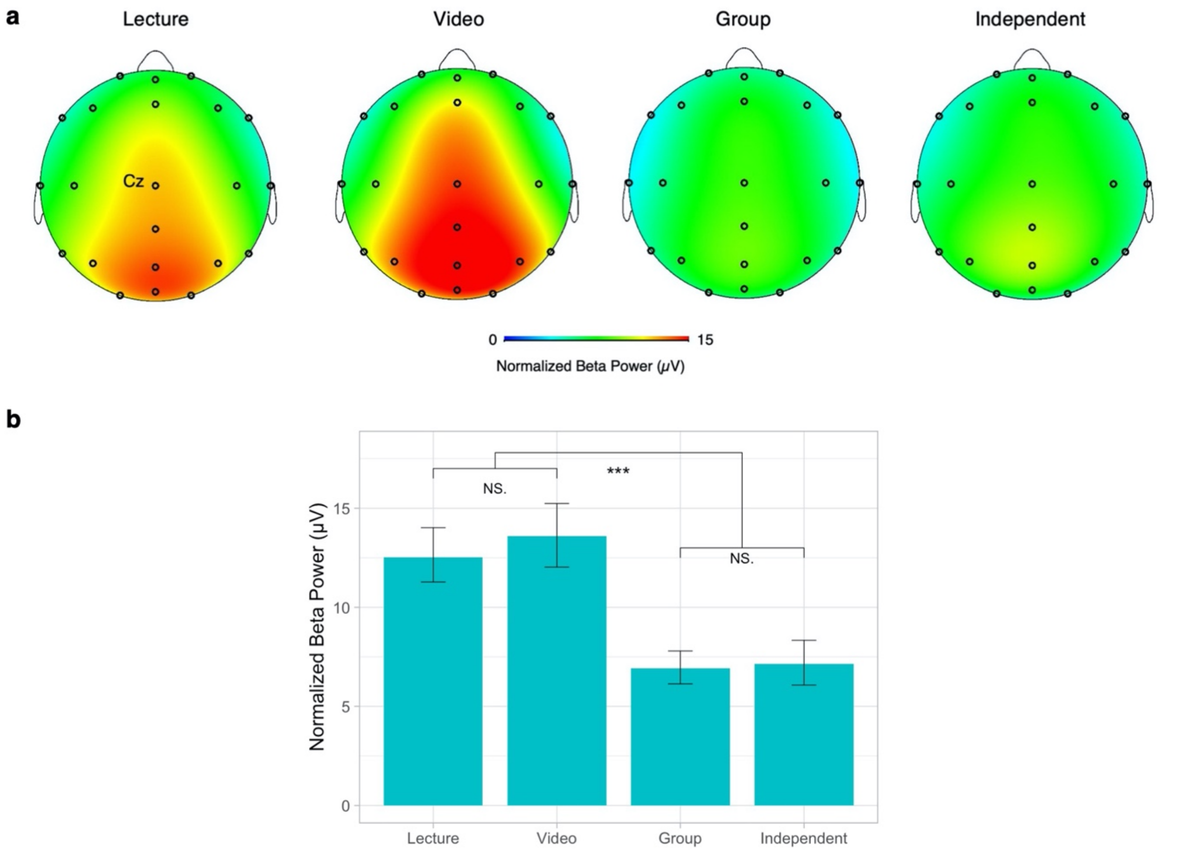

Supplementary Fig. 7: Effects of class activities on beta power (13-30hz), normalized by gamma power (30-50hz). **a** Topographic maps of beta band using gamma normalization. **b** Histogram of mean beta power in four activities, with error bars representing 95% confident interval (calculated by bootstrapping).

Results showed that instructional activity had a significant effect on beta power, $\chi^2(3) = 57.72$, $p < .001$. Planned contrasts revealed that beta power was significantly higher in teacher-initiated activities (lecture and video watching) than in student-initiated activities (group work and independent work), b = -3.11, t (55) = -9.75, $p < .001$. No difference was found in beta power between lecture and video, nor between group work and independent work (*p*s > .1).

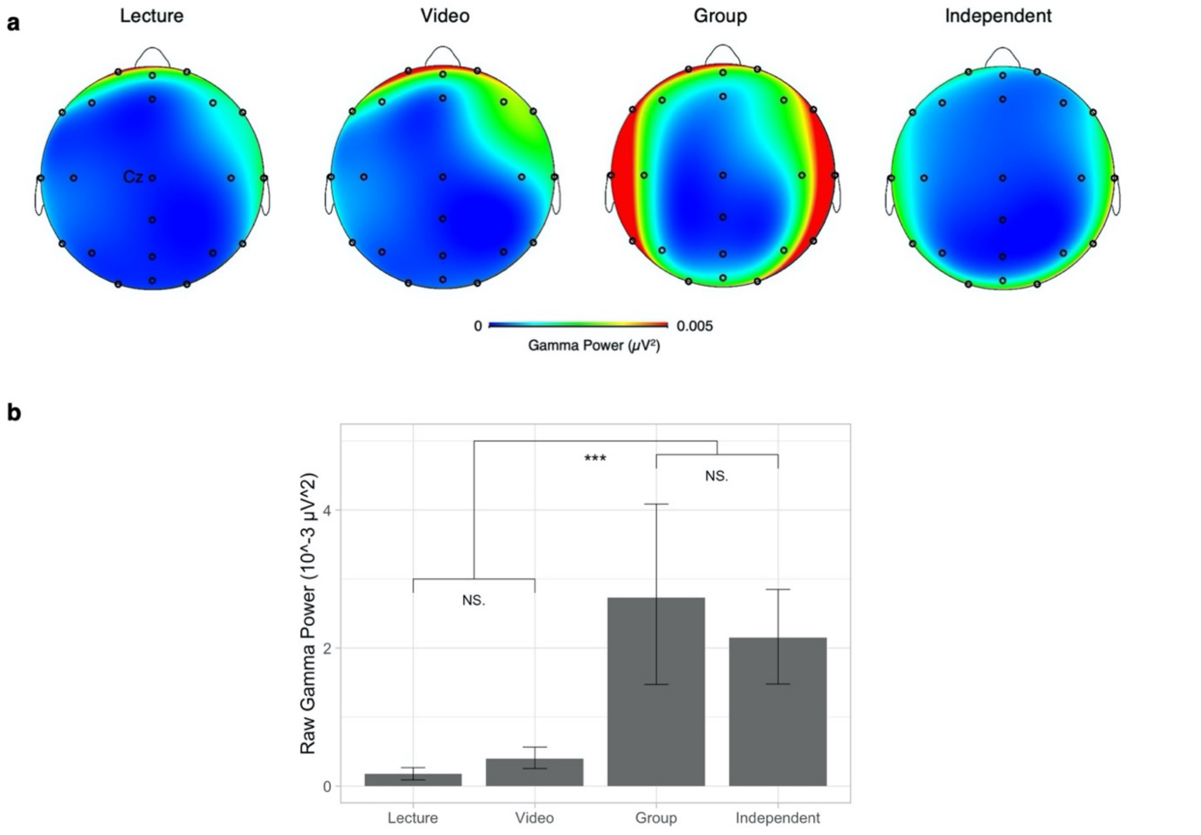

Supplementary Fig. 8: Effects of class activities on raw gamma power (30.5-50hz). **a** Topographic maps of non-normalized gamma band. **b** Histogram of mean non-normalized gamma power in four activities, with error bars representing 95% confident interval (calculated by bootstrapping).

Given that a normalization of 30-50hz entirely overlapped with the bandwidth for calculating gamma, results for gamma band using this normalization method become meaningless. Instead, we report the results from raw, non-normalized gamma power here. Results showed that instructional activity had a significant effect on gamma power, $\chi^2(3) = 17.23$, $p < .001$. Planned contrasts revealed that gamma power was significantly higher in student-initiated activities (group work and independent work) than in teacher-initiated activities (lecture and video watching), b = .001, t (55) = 4.32, $p < .001$. No difference was found in gamma power between lecture and video ($p = .76$), nor between discussion and independent work ($p = .43$).

Supplementary Figure 9: Spectrum plot of 1hz to 30hz after gamma normalization.

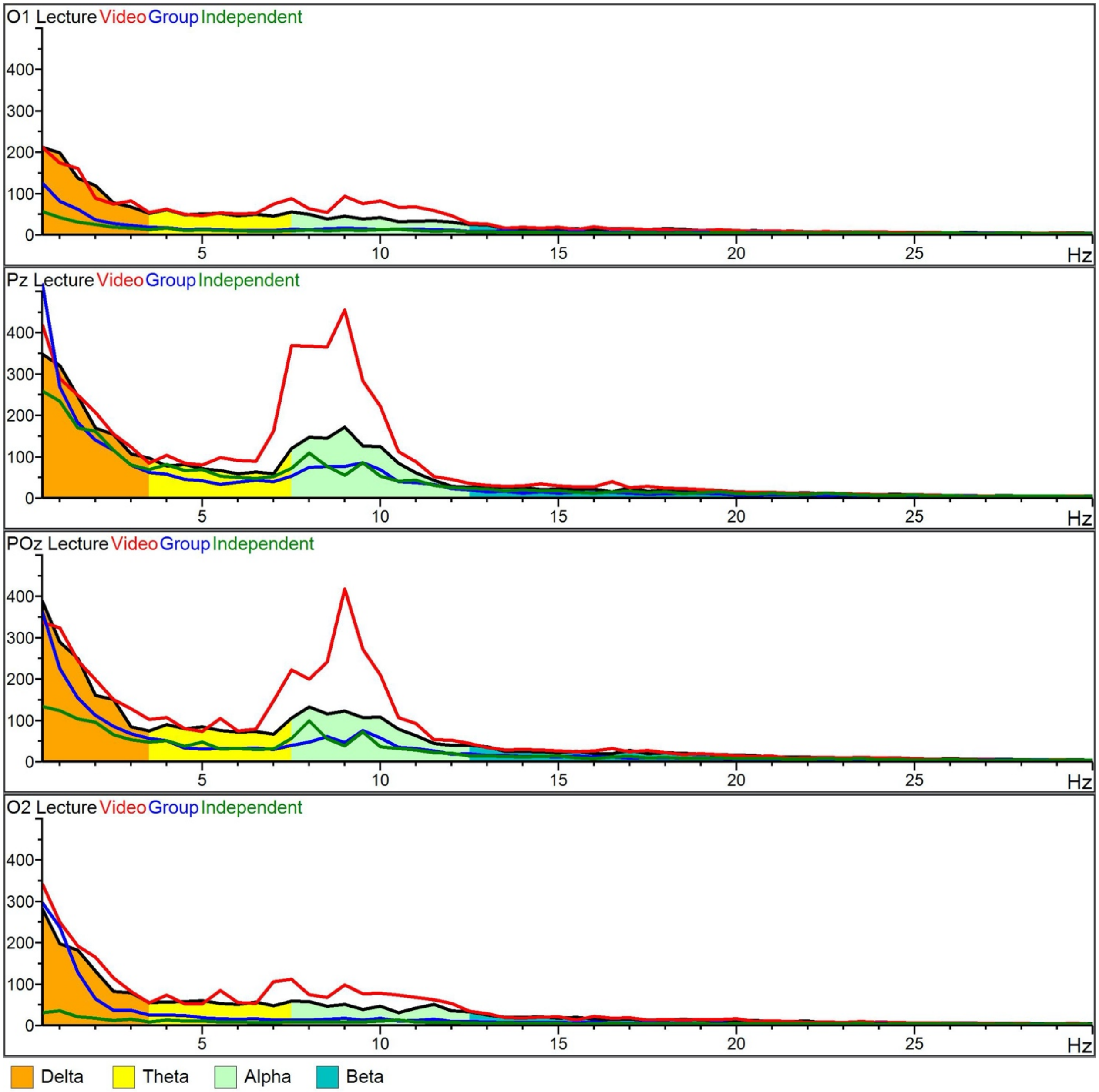

Supplementary Figure 10: Spectrum plot for raw gamma power.

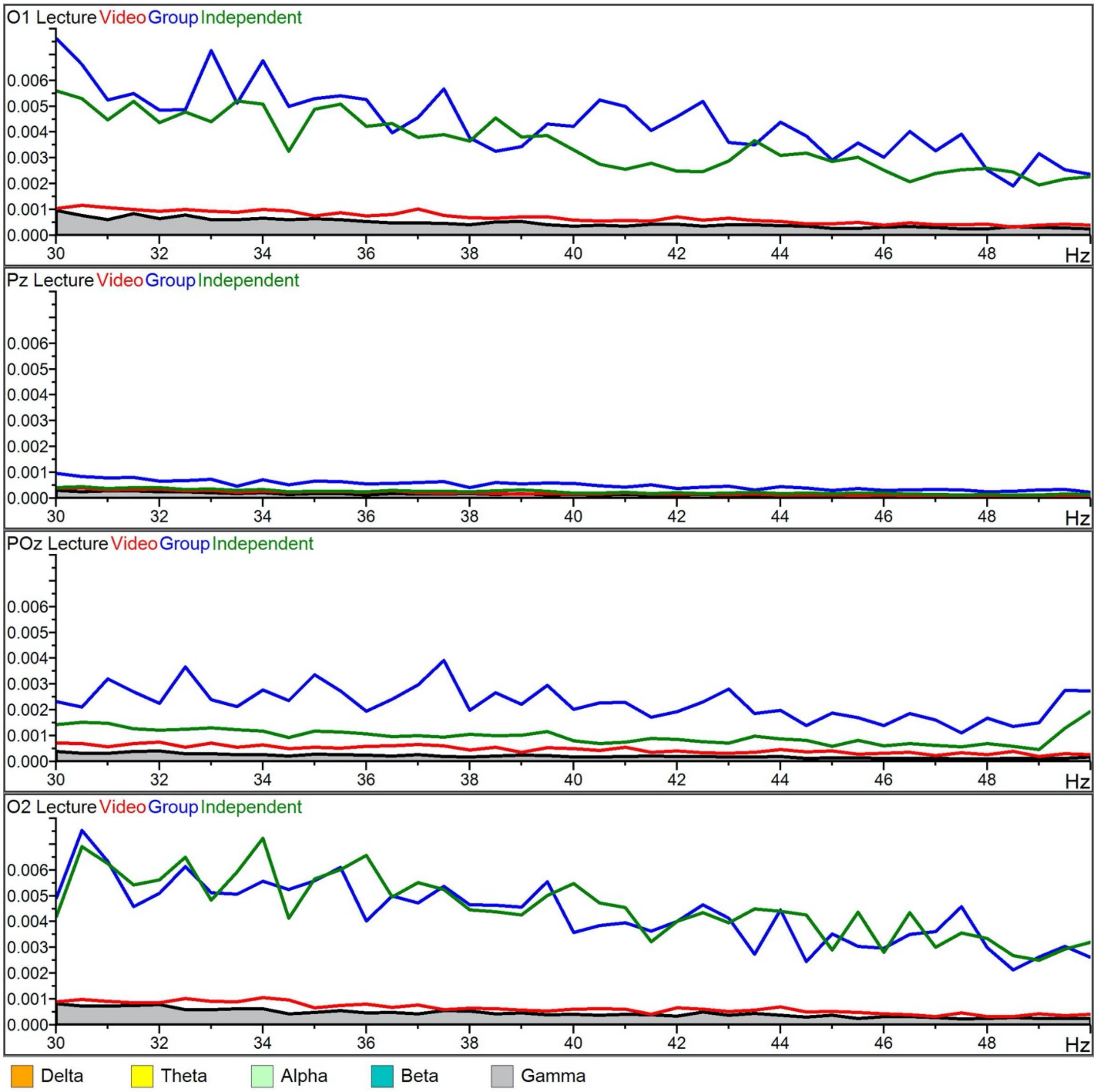

Supplement: Supplementary file 1 — Supplementary Information [file 41539_2021_94_MOESM1_ESM.pdf]
