# Supplementary material for: Effects of context on the neural correlates of attention in a college classroom
Source: NPJ Sci Learn. 2021 Jul 6;6:15. doi: 10.1038/s41539-021-00094-8 (PMC8260629; doi:10.1038/s41539-021-00094-8)
Supplement: Supplementary file 2 — Reporting Summary [file 41539_2021_94_MOESM2_ESM.pdf]

nature research

Corresponding author(s): Grammer

Last updated by author(s): Sep 29, 2020

Reporting Summary

Nature Research wishes to improve the reproducibility of the work that we publish. This form provides structure for consistency and transparency in reporting. For further information on Nature Research policies, see our Editorial Policies and the Editorial Policy Checklist.

Statistics

For all statistical analyses, confirm that the following items are present in the figure legend, table legend, main text, or Methods section.

n/a	Confirmed	
☐	☒	The exact sample size (*n*) for each experimental group/condition, given as a discrete number and unit of measurement
☐	☒	A statement on whether measurements were taken from distinct samples or whether the same sample was measured repeatedly
☐	☒	The statistical test(s) used AND whether they are one- or two-sided<br>*Only common tests should be described solely by name; describe more complex techniques in the Methods section.*
☐	☒	A description of all covariates tested
☐	☒	A description of any assumptions or corrections, such as tests of normality and adjustment for multiple comparisons
☐	☒	A full description of the statistical parameters including central tendency (e.g. means) or other basic estimates (e.g. regression coefficient) AND variation (e.g. standard deviation) or associated estimates of uncertainty (e.g. confidence intervals)
☐	☒	For null hypothesis testing, the test statistic (e.g. F, t, r) with confidence intervals, effect sizes, degrees of freedom and P value noted<br>*Give P values as exact values whenever suitable.*
☒	☐	For Bayesian analysis, information on the choice of priors and Markov chain Monte Carlo settings
☒	☐	For hierarchical and complex designs, identification of the appropriate level for tests and full reporting of outcomes
☐	☒	Estimates of effect sizes (e.g. Cohen's *d*, Pearson's *r*), indicating how they were calculated

Our web collection on statistics for biologists contains articles on many of the points above.

Software and code

Policy information about availability of computer code

Data collection	SMARTING mobile EEG
Data analysis	R, Brain Vision Analyzer

For manuscripts utilizing custom algorithms or software that are central to the research but not yet described in published literature, software must be made available to editors and reviewers. We strongly encourage code deposition in a community repository (e.g. GitHub). See the Nature Research guidelines for submitting code & software for further information.

Data

Policy information about availability of data

All manuscripts must include a data availability statement. This statement should provide the following information, where applicable:

- Accession codes, unique identifiers, or web links for publicly available datasets
- A list of figures that have associated raw data
- A description of any restrictions on data availability

The data that support the findings of this study are available from the corresponding author upon request.

Field-specific reporting

Please select the one below that is the best fit for your research. If you are not sure, read the appropriate sections before making your selection.

☐ Life sciences ☒ Behavioural & social sciences ☐ Ecological, evolutionary & environmental sciences

For a reference copy of the document with all sections, see nature.com/documents/nr-reporting-summary-flat.pdf

Behavioural & social sciences study design

All studies must disclose on these points even when the disclosure is negative.

Study description	Quantitative, EEG and behavior
Research sample	A diverse group of 23 healthy college students (Nmen = 5, ages 18 to 23 years-old) from a large public university participated (35% White, 17% Latinx, 39% Asian, and 8% other). Six participants reported speaking another language than English growing up. The breakdown in terms of gender and race ethnicity is reflective of the population from which the participants were recruited.
Sampling strategy	Students were recruited from a participant pool through the department of psychology. Upon being recruited, participants were randomly selected (based on random number generator) to participate in the EEG portion of the experiment.
Data collection	Data were collected in a college classroom where students participated in a lesson on educational neuroscience taught by a graduate teaching assistant. Students participated in groups of 4-9. Of these, 2-3 were randomly selected to be assessed with portable EEG. Lessons consisted of four 15-min instructional activities reflective of those common in a college course, taught in the following order: 1) whole-group lecture, 2) video watching, 3) group discussion, and 4) independent work. Sociodemographic data were collected for all of the participants and lessons were video-recorded.
Timing	Data were collected in the fall and winter terms of the 2019-2020 academic year.
Data exclusions	All data collected are described in the manuscript.
Non-participation	No participants declined participation or dropped out of the study.
Randomization	Participants were randomly selected to participate in the EEG component of the investigation based on random number generator. However, all comparisons described here are within subject.

Reporting for specific materials, systems and methods

We require information from authors about some types of materials, experimental systems and methods used in many studies. Here, indicate whether each material, system or method listed is relevant to your study. If you are not sure if a list item applies to your research, read the appropriate section before selecting a response.

Materials & experimental systems

n/a	Involved in the study	
☒	☐	Antibodies
☒	☐	Eukaryotic cell lines
☒	☐	Palaeontology and archaeology
☒	☐	Animals and other organisms
☒	☐	Human research participants
☒	☐	Clinical data
☒	☐	Dual use research of concern

Methods

n/a	Involved in the study	
☒	☐	ChIP-seq
☒	☐	Flow cytometry
☒	☐	MRI-based neuroimaging
